# Proanthocyanidin offers protection against diabetic nephropathy: elucidation of its mechanism of action using animal models

**DOI:** 10.1080/13880209.2024.2409772

**Published:** 2024-10-06

**Authors:** Dengpiao Xie, Huan Wang, Qing Ji, Jianting Wang

**Affiliations:** a Hospital of Chengdu University of Traditional Chinese Medicine, Chengdu, China; b Chengdu First People’s Hospital, Chengdu, China; c The Affiliated People’s Hospital of Fujian University of Traditional Chinese Medicine, Fuzhou, China

**Keywords:** Kidney injury, preclinical study, meta-analysis

## Abstract

**Context:**

Diabetic nephropathy (DN) is a major complication of diabetes mellitus and is the leading cause of kidney disease in patients undergoing renal replacement therapy. DN is associated with an increased risk of death in patients with diabetes. Conventional therapy for DN includes intensive control of blood glucose level and blood pressure and renin–angiotensin system blockade. However, this approach has limited treatment effects on DN. Therefore, identifying novel drugs to delay the progression of DN is urgently needed. Proanthocyanidin (PA) has been shown to exert potentially beneficial effects on DN. However, the protective mechanism and efficacy are yet to be elucidated.

**Objective:**

This study evaluates the efficacy and potential mechanisms of PA in animal models of DN.

**Methods:**

Preclinical studies were searched from Chinese National Knowledge Infrastructure, PubMed, Web of Science, Embase, and Google Scholar databases, with the search deadline of August 2023. Keywords (‘diabetic nephropathies’, ‘nephropathies, diabetic’, ‘diabetic kidney diseases’, ‘proanthocyanidin’, ‘anthocyanidin polymers’, ‘procyanidins’, ‘animal*’, ‘rat’, and ‘mice’) were used to search the databases. RevMan 5.3 was used for statistical analysis.

**Results:**

A total of 22 studies involving 538 animals were included in this analysis. The pooled results indicated that PA therapy significantly improved kidney function and reduced proteinuria and blood glucose levels. The protective mechanism of PA was associated with anti-inflammatory, antioxidant, antifibrotic, and antiapoptotic effects; inhibition of endoplasmic reticulum stress; and alleviation of mitochondrial dysfunction and dyslipidemia.

**Conclusion:**

These findings suggest that PA alleviates DN by mediating multiple targets and pathways.

## Introduction

Diabetic nephropathy (DN) is a severe complication of diabetes mellitus and is characterized by the presence of proteinuria, diabetic glomerular lesions, and gradually reduced kidney function. As the disease progresses, in its late stage, the mesangial matrix accumulates in the kidney, and glomerular fibrosis occurs (Qi et al. 2017). The prevalence of DN in patients with diabetes ranges from 20% to 40% (Shahbazian and Rezaii 2013). In China, the incidence of DN has increased drastically in the past few decades. A study has estimated that patients with diabetes-induced chronic kidney disease (CKD) exceeded 24 million in 2015 (Zhang et al. 2016). DN has become the major cause of end-stage renal disease (ESRD) in developed countries such as the United States. Furthermore, DN is linked to an increased risk of all-cause and cardiovascular death in patients with CKD (Afkarian et al. 2013), imposing a heavy humanistic and economic burden globally. Conventional treatment for DN includes intensive blood glucose and blood pressure control and the administration of renin–angiotensin–aldosterone and sodium–glucose cotransporter inhibitors. In addition, novel therapeutic strategies have been developed to manage DN. For instance, mineralocorticoid receptor antagonists and endothelin receptor antagonists have been reported to exhibit anti-inflammatory and antifibrotic effects in DN. Clinical trials on drugs for DN, such as spironolactone, eplerenone, and avosentan, have shown that these can significantly reduce proteinuria and delay the decrease in renal function (Mann et al. 2010; Bakris et al. 2015). Other drugs targeting inflammation, free radicals, and Nrf2 activators have also been reported to exert potential therapeutic effects on DN. However, high-quality clinical trials are required to confirm their efficacy (Samsu 2021). Apart from these drugs, natural bioactive components in plants can be used to treat DN. Meta-analysis of animal studies indicated that resveratrol, quercetin, and ginsenosides can enhance kidney function, reduce proteinuria by inhibiting inflammation, exert antioxidant activities, and also act *via* other mechanisms (Hu et al. 2022; Hu et al. 2022; Chen et al. 2023). Nonetheless, clinical trials to prove the effects of these bioactive components in patients with DN are lacking. Moreover, although these approaches can delay the progression of DN, the number of patients with diabetes who develop CKD and progress to ESRD is increasing. Therefore, novel drugs to prevent or treat DN must be identified.

**Figure 1.**
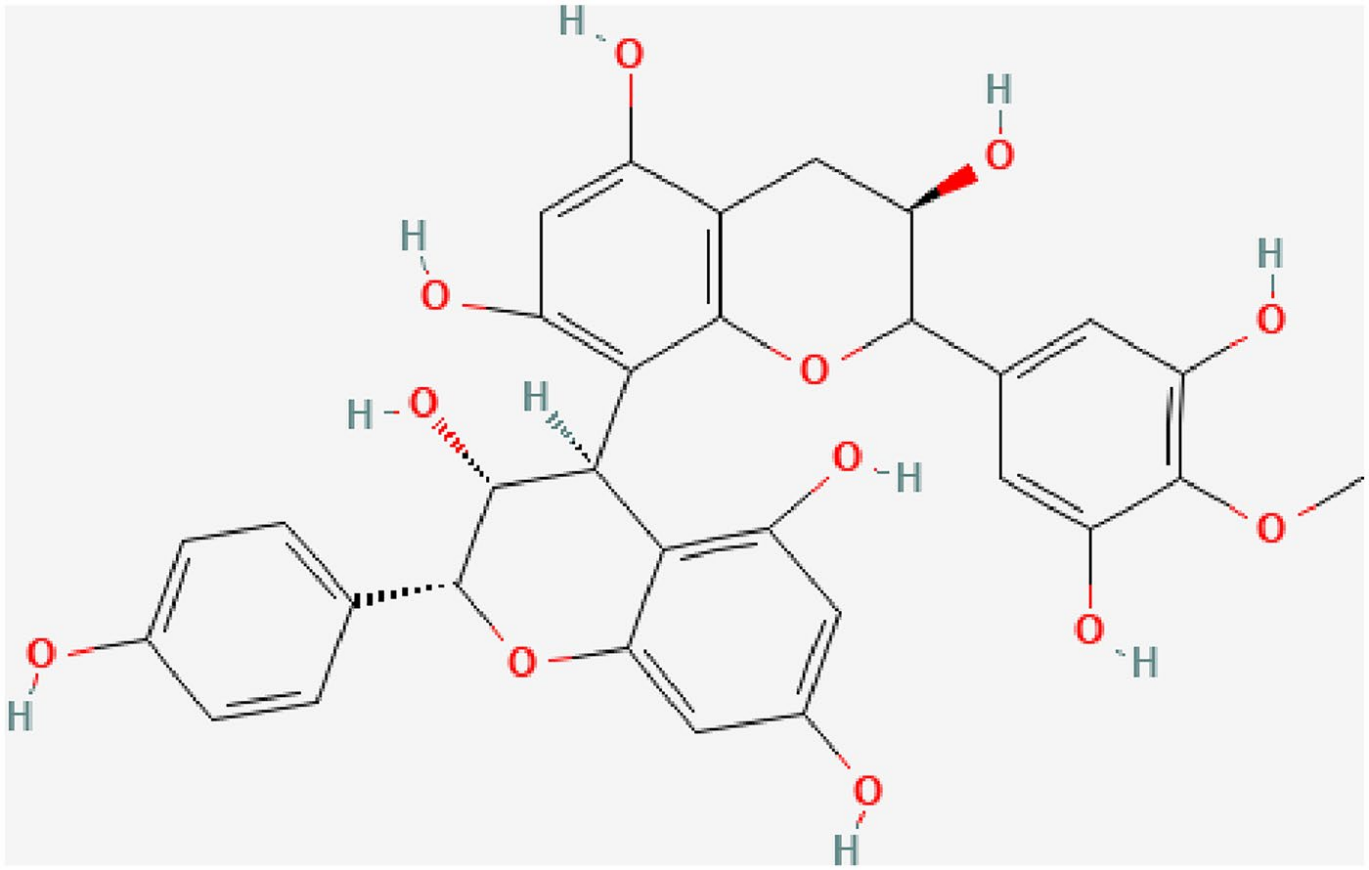
Chemical structure of proanthocyanidin.

The use of natural plants and their bioactive components has become a potential therapeutic strategy for DN. Compared with conventional drugs, these can be developed more easily and might exert pleiotropic effects, thus accentuating the treatment effects. Proanthocyanidin (PA) is a class of polyphenolic compounds rich in certain flowers, nuts, fruits, and seeds such as grapes (Rauf et al. 2019). The structure of PA is illustrated in Figure 1. PA has been demonstrated to possess various pharmacological properties, including antioxidant, antiapoptotic, anti-inflammatory, cardiovascular protective, anticancer, and immunomodulatory effects (Rauf et al. 2019). Furthermore, PA has been reported to show potent protective effects in diabetes and its complications in both *in vitro* and *in vivo* studies (Sun et al. 2016). In addition, animal studies have implied that PA is one of the most promising drugs for DN therapy. However, these studies lack conclusive evidence, which limits their clinical translation.

Animal experiments are often performed to assess the effect of drugs before clinical trials and are valuable tools to explore the mechanisms and etiology of diseases. However, certain shortcomings exist in animal studies. For instance, the sample size is small, and hence, the statistical power is insufficient to assess the true effects. Moreover, a single animal study cannot reveal the complex pathophysiological mechanisms of diseases. Therefore, preclinical review of animal studies is important in drug development and the elucidation of pathophysiological mechanisms by researchers. To the best of our knowledge, preclinical systematic reviews are not available to determine the effects of PA on DN. Therefore, this study aimed to assess the protective effects and potential mechanisms of PA against DN in animal models by performing a systematic review.

## Methods

### Review protocol

This systematic review was performed and reported with reference to the Preferred Reporting Item for Systematic Reviews and Meta-analysis and the Systematic Review Centre for Laboratory Animal Experimentation.

### Data sources and search strategies

A comprehensive search was performed in Chinese National Knowledge Infrastructure, PubMed, Web of Science, Embase, and Google Scholar databases, with the search deadline no later than August 2023. The language was restricted to English and Chinese. Keywords (‘diabetic nephropathies’, ‘nephropathies, diabetic’, ‘diabetic kidney diseases’, ‘diabetic glomerulosclerosis’, ‘proanthocyanidin’, ‘condensed tannin’, ‘anthocyanidin polymers’, ‘procyanidins’, ‘animal*’, ‘rat’, and ‘mice’) were used to search the databases. Moreover, references to similar studies or reviews were searched for potential studies.

### Inclusion and exclusion criteria

The inclusion criteria were as follows: (1) DN models were established in animals without restricting the modeling methods. DN models were successfully established, as defined by significantly worse blood sugar levels, renal function, and proteinuria compared with the control group. (2) The treatment group received PA monotherapy (3) The model group received the same treatment regimen, except PA. (4) The primary outcomes were serum creatinine (Scr), blood urea nitrogen (BUN), and proteinuria. The secondary outcomes were fasting blood glucose level, body weight, inflammatory markers, oxidative stress markers, serum lipid levels, extracellular matrix (ECM), and other potential mechanisms of PA against DN.

The exclusion criteria were as follows: (1) *in vitro* studies; (2) other kidney injury models; (3) the treatment group received other interventions; (5) duplicate publication; (6) full-text not available; (7) reviews, case reports, and comments.

### Study selection, data extraction, and quality assessment

Eligible studies were screened in three steps. Each step was performed by two independent reviewers. First, ineligible studies were excluded based on the assessment of titles and abstracts of relevant studies. Second, full-text studies were downloaded and reviewed. Third, disagreements were resolved between the investigators *via* discussion, and if necessary, a third reviewer was consulted.

The data were extracted by two independent researchers. The following data were extracted into a spreadsheet: (1) name of the first author and year of publication; (2) characteristics of the animals, including the number of animals in the treatment and model groups, species, age, and sex; (3) methods for establishing the DN models; (4) information on PA treatments, including dose, route of administration, and treatment course; and (5) the index of primary and secondary outcomes. If the treatment group received various doses of PA therapy, all data were included in this meta-analysis for studying the dose effects. In this case, the number of animals in the model group was divided by the number of PA groups. When the data were expressed graphically, an attempt was made to contact the corresponding authors for detailed information, and if no response was received, the data were measured graphically using the digital ruler software. Mean and standard deviation (SD) were extracted from each study; however, if the data were expressed as standard error (SE), they were converted to SD using the appropriate formula (Altman and Bland 2005).

The study quality was determined in accordance with the modified Systematic Review Center for Laboratory Animal Experimentation risk of bias tool (Hooijmans et al. 2014). It included the following checklist: (1) peer-reviewed publication; (2) randomization of the animals to the treatment or model group; (3) baseline characteristics; (4) allocation concealment; (5) random housing; (6) blinding methods for animal breeders and researchers; (7) random outcome assessment; (8) blinding of outcome assessors; (9) incomplete outcome data; and (10) other sources of bias.

### Data synthesis and analysis

The mean difference (MD) with a 95% confidence interval (CI) was calculated for the pooled results when all studies measured the outcomes on the same scale. On the contrary, when the outcomes were reported on different scales, standardized mean difference (SMD) with CI was used to compute the pooled effects. The *I*^2^ test was used to evaluate the heterogeneity and an *I*^2^ value of <25% was regarded as low heterogeneity. Values between 25% and 50% were regarded as moderate heterogeneity, and those >50% suggested substantial heterogeneity. Random-effects and fixed-effects models were applied to the pooled results with *I*^2^ > 50% or I^2^ ≤ 50%, respectively.

### Additional analysis and publication bias

To ascertain the effects of certain factors that could influence the pooled effects of primary outcomes, subgroup analysis was performed based on the animal species (rats, mice) and methods for establishing the DN models (streptozotocin (STZ), db/db). A funnel plot of primary outcomes was constructed to assess potential publication bias.

## Results

### Study selection and characteristics

**Figure 2.**
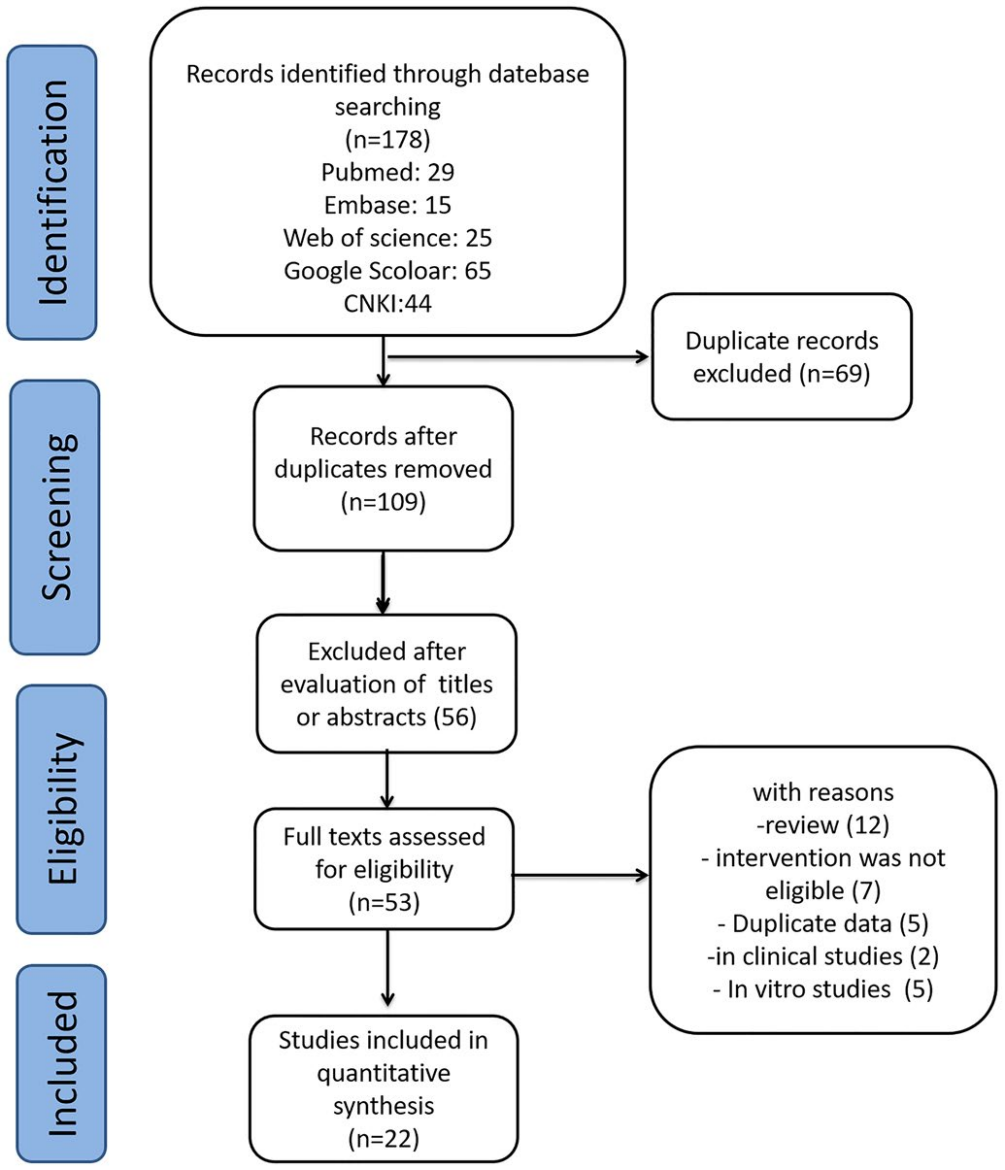
The selection flow of eligible studies in the meta-analysis.

A total of 178 potentially relevant studies were included, of which 69 duplicate studies were eliminated. The remaining 109 studies were further reviewed based on the title and abstract, and 56 studies were excluded. The full texts of 53 studies were carefully reviewed, of which 31 studies were excluded for the following reasons: (1) reviews; (2) intervention was not eligible; (3) duplicate publication; (4) *in vitro* studies. Finally, 22 studies were included in the systematic review (Liu et al. 2006; Lee et al. 2007; Li et al. 2008; Li et al. 2009; Liu et al. 2009; Mansouri et al. 2011, 2015; Sayed 2012; Al-Malki et al. 2013; Bao et al. 2014; Wang 2016; Du et al. 2017; Salahuddin and Katary 2017; Wei et al. 2017, 2018; Gao et al. 2018; Gong et al. 2018, 2021; Aziza et al. 2019; Ding et al. 2020; Lv et al. 2021; Gao et al. 2022), and the selection flow is represented in Figure 2.

The study characteristics are summarized in Table 1. A total of 22 studies were included in this review. A total of 538 animals were involved, of which 310 were in the PA group and 228 were in the model group. Eight studies were published in Chinese (Liu et al. 2006; Liu et al. 2009; Wang 2016; Du et al. 2017; Wei et al. 2017; Gong et al. 2018; Lv et al. 2021; Gao et al. 2022) and 14 in English (Lee et al. 2007; Li et al. 2008; Li et al. 2009; Mansouri et al. 2011, 2015; Sayed 2012; Al-Malki et al. 2013; Bao et al. 2014; Salahuddin and Katary 2017; Gao et al. 2018; Wei et al. 2018; Aziza et al. 2019; Ding et al. 2020; Gong et al. 2021). Of the 22 studies, one study used apoE ^−/−^ on C57BL/6J genetic background mice (Al-Malki et al. 2013), three utilized db/db mice (Du et al. 2017; Wei et al. 2017, 2018), one used Kunming mice (Gong et al. 2021), three used albino rats (Sayed 2012; Salahuddin and Katary 2017; Aziza et al. 2019), ten used Sprague–Dawley rats (Liu et al. 2006; Mansouri et al. 2011, 2015; Bao et al. 2014; Wang 2016; Gao et al. 2018; Gong et al. 2018; Ding et al. 2020; Lv et al. 2021; Gao et al. 2022), and five used Wistar rats (Lee et al. 2007; Li et al. 2008; Li et al. 2009; Liu et al. 2009; Salahuddin and Katary 2017). Male animals were used in 20 studies (Liu et al. 2006; Lee et al. 2007; Li et al. 2008; Li et al. 2009; Liu et al. 2009; Bei et al. 2011; Mansouri et al. 2011, 2015; Sayed 2012; Al-Malki et al. 2013; Bao et al. 2014; Du et al. 2017; Salahuddin and Katary 2017; Wei et al. 2017, 2018; Gao et al. 2018; Gong et al. 2018, 2021; Aziza et al. 2019; Lv et al. 2021; Gao et al. 2022), and two studies did not report the sex of the animals (Wang 2016; Ding et al. 2020). Regarding the methods for establishing DN animal models, STZ-induced DN models were used in 17 studies (Lee et al. 2007; Li et al. 2008; Li et al. 2009; Liu et al. 2009; Mansouri et al. 2011, 2015; Sayed 2012; Al-Malki et al. 2013; Bao et al. 2014; Wang 2016; Salahuddin and Katary 2017; Gao et al. 2018; Gong et al. 2018; Aziza et al. 2019; Ding et al. 2020; Lv et al. 2021; Gao et al. 2022), db/db DN models in three studies (Du et al. 2017; Wei et al. 2017, 2018), CdCl_2_-induced DN model in one study (Gong et al. 2021), and alloxan-induced DN model in one study (Liu et al. 2006). The PA dose was in the range of 5–500 mg/kg body weight. Five studies used more than one dose of PA (Liu et al. 2006; Lee et al. 2007; Bao et al. 2014; Ding et al. 2020; Gao et al. 2022), and the low, medium, and high doses were marked as a, b, and c, respectively, in the pooled results. The treatment duration ranged from 20 d to 24 weeks. PA was administered orally in 20 studies (Liu et al. 2006; Lee et al. 2007; Li et al. 2008; Li et al. 2009; Liu et al. 2009; Mansouri et al. 2011, 2015; Sayed 2012; Al-Malki et al. 2013; Bao et al. 2014; Wang 2016; Du et al. 2017; Salahuddin and Katary 2017; Wei et al. 2017, 2018; Gao et al. 2018; Aziza et al. 2019; Ding et al. 2020; Lv et al. 2021; Gao et al. 2022) and *via* intraperitoneal injection in 2 studies (Gong et al. 2018, 2021).

**Table 1.** Basic characteristics of studies.

Study	Species	Age	*N* = treatment/Model	Model Establishment	Treatment group	Treatment duration
Al-Malki et al. (2013)	Male apoE^−/−^ on C57BL/6J genetic background mice	14 weeks	5/5	Mice were i.p. injected with 40 mg/kg STZ at a dose of bodyweight and fed with a high-fat diet.	Mice were fed with a high-fat diet containing 500 mg/kg PA.	12 weeks
Aziza et al. (2019)	Male albino rats	5–6 weeks	18/24	Rats were i.p. injected with STZ at a dose of 50 mg/kg bodyweight	Rats were treated daily with PA (250 mg/kg body weight orally)	6 weeks
Bao et al. 2014	Male Sprague-Dawley rats	6 weeks	36/12	Rats were fed on high-carbohydrate/high-fat diet for 4 weeks, then i.p. injected with 30 mg/kg STZ of bodyweight. Another STZ iniection was repeated after 7 d.	Rats were treated orally with low PA (125 mg/kg body weight), medium PA (250 mg/kg body weight), and high PA (500 mg/kg body weight)	16 weeks
Ding et al. 2020	Sprague-Dawley rats	–	20/10	Rats were fed high-sugar and high-lipid fodder for 4 weeks. Then rats were i.p. injected with 40 mg/kg STZ of bodyweight (two injections, with a 48 h interval).	Rats were orally administered with 125 mg/kg/d or 250 mg/kg/d PA.	8 weeks
Du et al. (2017)	Male db/db mice	6 weeks	8/8	db/db diabetic model	Mice were orally administered with 5 mg/kg/d PA.	8 weeks
Gao et al. (2018**)**	Male Sprague-Dawley rats	8–12 weeks	15/16	Rats were fed on high-fat diet for one month, then i.p. injected with 40 mg/kg STZ of bodyweight.	Rats were orally administered with 250 mg/kg/d PA.	16 weeks
Gao et al. (2022)	Male Sprague-Dawley rats	–	36/12	Rats were i.p. injected with 55 mg/kg STZ at a dose of bodyweight	Rats were treated orally with low PA (50 mg/kg body weight), medium PA (100 mg/kg body weight), and high PA (150 mg/kg body weight)	16 weeks
Gong et al. (2018)	Male Sprague-Dawley rats	–	8/8	Rats were i.p. injected with 30 mg/kg STZ of bodyweight. Another STZ injection was repeated after 7 d.	Rats were i.p. injected with 150 mg/kg/d PA	4 weeks
Gong et al. (2021)	Male Kunming mice	5 weeks	10/10	Rats were fed on high-fat and high-sugar diet and i.p. injected with 1 mg/kg Cdcl_2_ of bodyweight for 12 weeks	Rats were i.p. injected with 5 mg/kg/d PA	4 weeks
Lee et al. (2007)	Male Wistar rats	–	14/7	Rats were i.p. injected with STZ at a dose of 50 mg/kg of body weight	Rats were orally administered polymers or oligomers at a dose of 10 mg/kg body weight/day.	20 d
Li et al. (2008)	Male Wistar rats	10 weeks	13/9	Rats received tail vein injection of STZ at a dose of 55 mg/kg of body weight	Rats were orally administered with 250 mg/kg/d PA.	24 weeks
Li et al. (2009)	Male Wistar rats	–	10/8	Rats injected with STZ	Rats were administered with 500 mg/kg/d PA	24 weeks
Liu et al. (2006**)**	Male Sprague-Dawley rats	–	20/10	Rats were i.p. injected with 200 mg/kg alloxan of bodyweight. Another Alloxan injection was repeated after 24 h.	Rats were orally administered with 50 mg/kg/d or 150 mg/kg/d PA.	6 weeks
Liu et al. (2009)	Male Wistar rats	3 months	10/8	Rats were fed on high-fat diet for 4 weeks, then i.p. injected with 25 mg/kg STZ of bodyweight.	Rats were orally administered with 200 mg/kg/d PA.	12 weeks
Lv et al. (2021**)**	Male Sprague-Dawley rats	5 weeks	10/10	Rats were fed on high-fat diet for 4 weeks, then i.p. injected with 50 mg/kg STZ of bodyweight.	Rats were orally administered with 500 mg/kg/day PA.	12 weeks
Mansouri et al. (2011)	Male Sprague-Dawley rats	–	10/10	Rats were i.p. injected with STZ at a dose of 50 mg/kg bodyweight.	Rats were orally administered with 500 mg/kg/d PA	6 weeks
Mansouri et al. (2015)	Male Sprague-Dawley rats	–	6/6	Rats were i.p. injected with STZ at a dose of 50 mg/kg bodyweight.	Rats were orally administered with 200 mg/kg/d PA	4 weeks
Salahuddin and Katary (2017)	Male albino Wistar rats	–	15/15	Rats were i.p. injected with STZ at a dose of 60 mg/kg bodyweight.	Rats were orally administered with 200 mg/kg/d PA	8 weeks
Sayed (2012)	Male albino rats	9 weeks	8/12	Rats received tail vein injection of STZ at a dose of 65 mg/kg of body weight	Rats were orally administered with 250 mg/kg/d PA	12 weeks
Wang (2016)	Sprague-Dawley rats	3 months	20/10	Rats were i.p. injected with STZ at a dose of 60 mg/kg bodyweight.	Rats were orally administered with 200 mg/kg/d or 400 mg/kg/d PA.	8 weeks
Wei et al. (2017)	Male C57Bl db/db mice	6–8 weeks	8/8	db/db diabetic model	Mice were orally administered with 5 mg/kg/d PA.	12 weeks
Wei et al. (2018)	Male C57Bl db/db mice	6–8 weeks	10/10	db/db diabetic model	Mice were orally administered with 30 mg/kg/d PA.	12 weeks

### Primary outcomes

#### Scr

**Figure 3.**
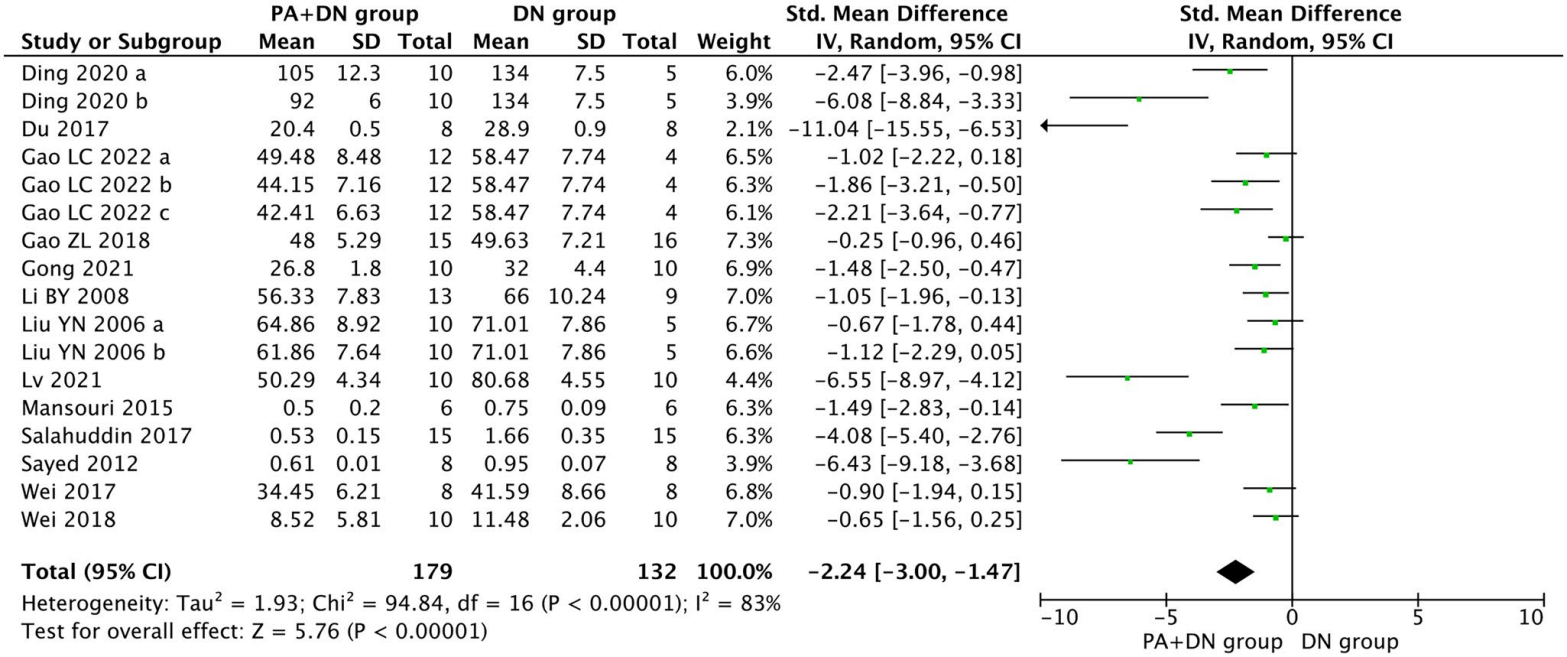
Forest Plot depicting the effect of PA on scr.

Scr was reported in 13 studies (Liu et al. 2006; Li et al. 2009; Mansouri et al. 2011, 2015; Bao et al. 2014; Wang 2016; Du et al. 2017; Salahuddin and Katary 2017; Wei et al. 2017, 2018; Gao et al. 2018; Gong et al. 2018; Ding et al. 2020; Lv et al. 2021). In the pooled analysis, compared with the model group, Scr was significantly reduced in the group treated with PA (SMD, −2.24; 95% CI, −3.00 to −1.47; *p* < 0.00001; heterogeneity: I^2^ = 83%, Figure 3).

#### Bun

**Figure 4.**
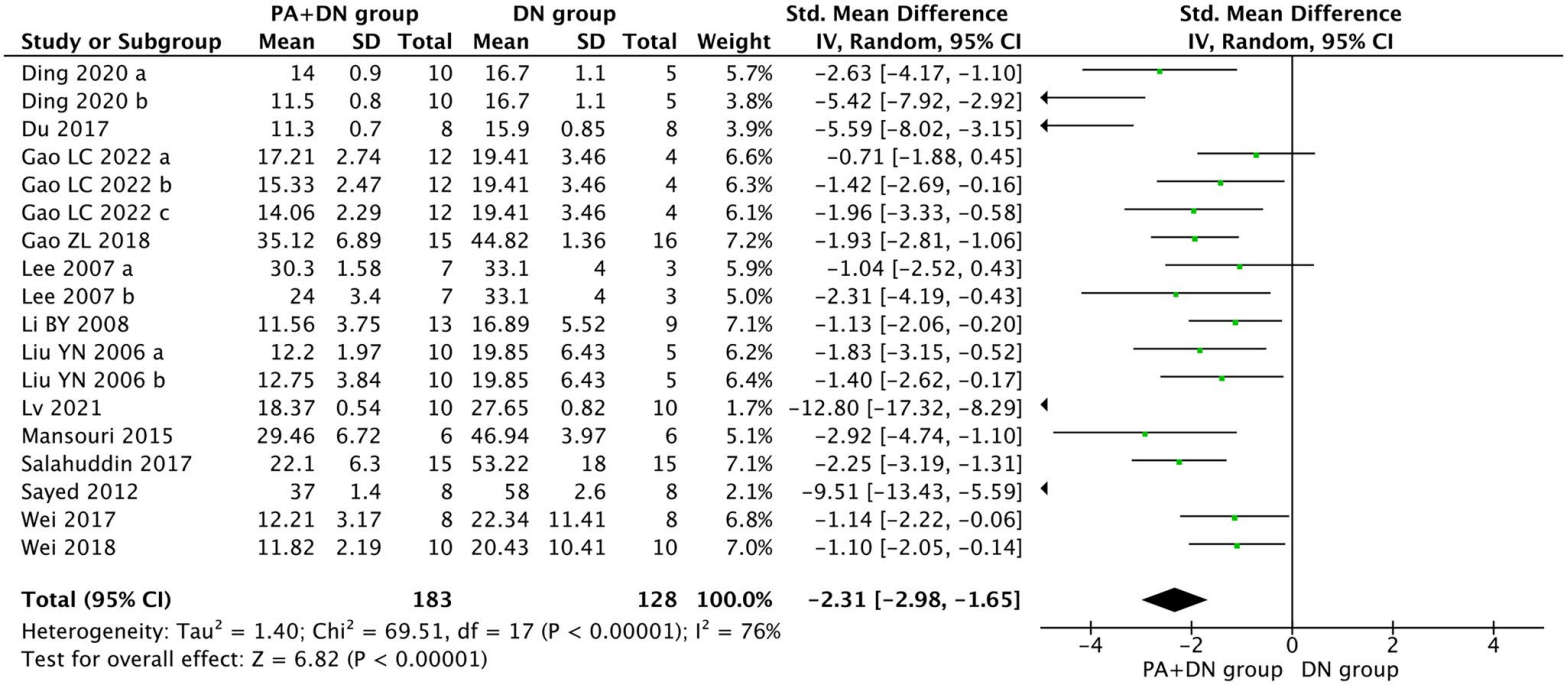
Forest Plot depicting the effect of PA on BUN.

BUN was reported in 13 studies (Liu et al. 2006; Lee et al. 2007; Li et al. 2008; Sayed 2012; Mansouri et al. 2015; Du et al. 2017; Salahuddin and Katary 2017; Wei et al. 2017, 2018; Gao et al. 2018; Ding et al. 2020; Lv et al. 2021; Gao et al. 2022). In the pooled analysis, compared with the model group, BUN was significantly reduced in the group treated with PA (SMD, −2.31; 95% CI, −2.98 to −1.65; *p* < 0.00001; heterogeneity: I^2^ = 76%, Figure 4).

#### Proteinuria

**Figure 5.**
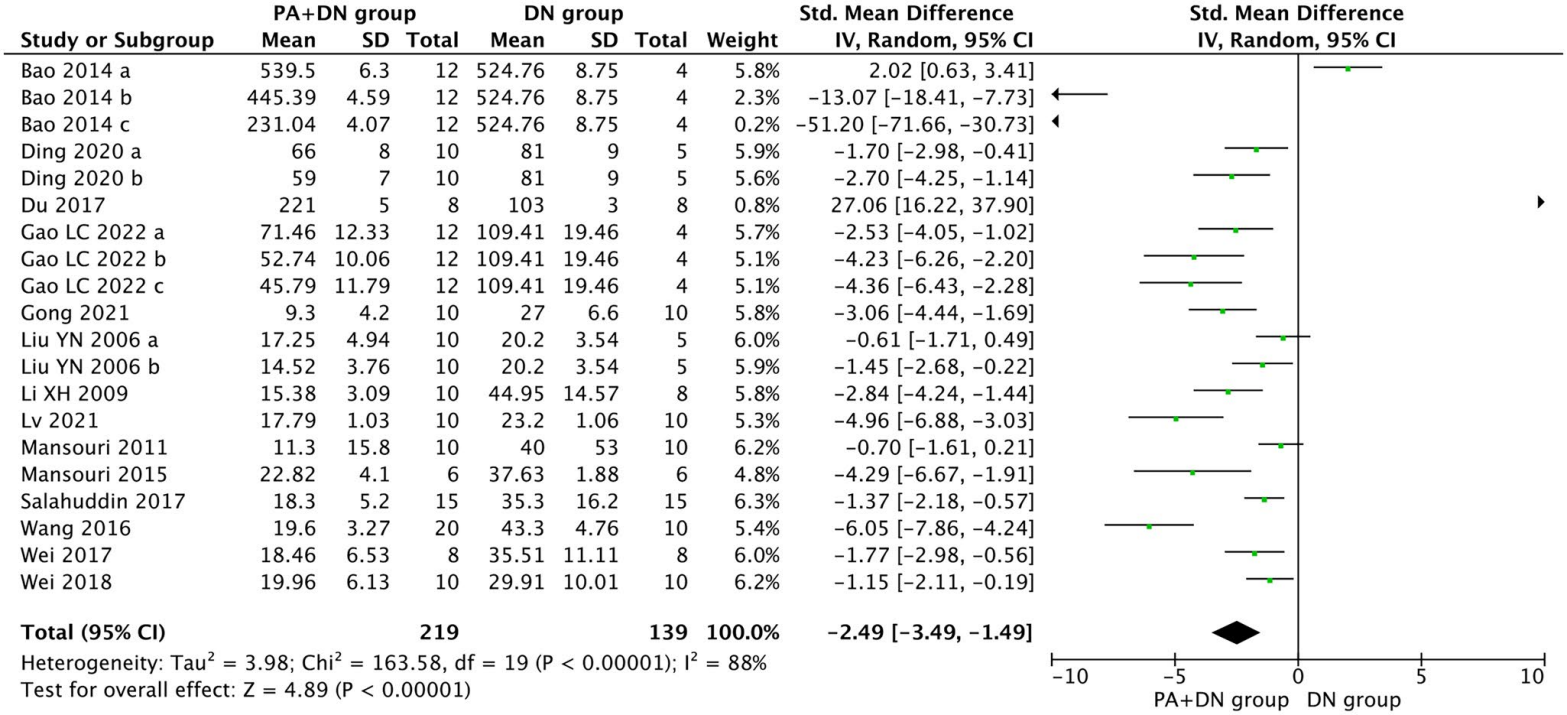
Forest Plot depicting the effect of PA on proteinuria.

Proteinuria was reported in 14 studies (Liu et al. 2006; Li et al. 2009; Mansouri et al. 2011, 2015; Bao et al. 2014; Wang 2016; Du et al. 2017; Salahuddin and Katary 2017; Wei et al. 2017, 2018; Ding et al. 2020; Gong et al. 2021; Lv et al. 2021; Gao et al. 2022). In the pooled analysis, compared with the model group, proteinuria was significantly reduced in the group treated with PA (SMD, −2.49; 95% CI, −3.49 to −1.49; *p* < 0.00001; heterogeneity: I^2^ = 88%, Figure 5).

### Secondary outcomes

#### ECM of glomeruli

In the pooled analysis of ECM of glomeruli, two studies were included in this review (Li et al. 2009; Gao et al. 2018). The pooled results demonstrated that compared with the model group, PA treatment significantly reduced the ECM of glomeruli (SMD, −9.84; 95% CI, −12.06 to −7.62; *p* < 0.00001; heterogeneity: I^2^ = 32%).

#### Fasting glucose and hemoglobin A1c

In the pooled analysis of fasting blood glucose levels, 20 studies were included in this review (Lee et al. 2007; Li et al. 2008; Li et al. 2009; Liu et al. 2009; Sayed 2012; Al-Malki et al. 2013; Bao et al. 2014; Mansouri et al. 2015; Wang 2016; Du et al. 2017; Salahuddin and Katary 2017; Wei et al. 2017, 2018; Gao et al. 2018; Gong et al. 2018, 2021; Aziza et al. 2019; Ding et al. 2020; Lv et al. 2021; Gao et al. 2022). The pooled analysis revealed that compared with the model group, PA significantly reduced the fasting blood glucose level (SMD, −3.71; 95% CI, −4.67 to −2.75; *p* < 0.00001; heterogeneity: I^2^ = 89%).

In the pooled analysis of hemoglobin A1c, four studies were included in this review (Lee et al. 2007; Li et al. 2008; Li et al. 2009; Al-Malki et al. 2013; Bao et al. 2014). The pooled results indicated that compared with the model group, PA significantly reduced hemoglobin A1C (SMD, −1.53; 95% CI, −2.26 to −0.81; *p* < 0.00001; heterogeneity: I^2^ = 57%).

#### Body weight, kidney weight, and kidney weight/body weight

In DN animal models, the pooled results showed that the administration of PA was associated with increased body weight (SMD, 2.23; 95% CI, 0.80–3.65; *p* < 0.00001; heterogeneity: I^2^ = 92%) in 10 studies (Li et al. 2008; Mansouri et al. 2011; Sayed 2012; Al-Malki et al. 2013; Bao et al. 2014; Du et al. 2017; Wei et al. 2018; Ding et al. 2020; Gong et al. 2021; Lv et al. 2021), decreased kidney weight (SMD, −1.54; 95% CI, −2.75 to −0.33; *p* = 0.01; heterogeneity: I^2^ = 74%) in 3 studies (Mansouri et al. 2011; Du et al. 2017; Ding et al. 2020), and decreased kidney weight/body weight (SMD, −3.18; 95% CI, −5.75 to −0.61; *p* < 0.00001; heterogeneity: I^2^ = 93%) in 4 studies (Li et al. 2008; Li et al. 2009; Liu et al. 2009; Bao et al. 2014).

#### Oxidative stress markers: glutathione (GSH), superoxide dismutase (SOD), catalase (CAT), and malondialdehyde (MDA)

The pooled results implied that PA significantly alleviated oxidative stress. In DN animal models, the pooled results indicated that PA significantly increased the GSH levels (SMD, 2.45; 95% CI, 1.38–3.51; *p* < 0.00001; heterogeneity: I^2^ = 67%) in 5 studies (Lee et al. 2007; Sayed 2012; Al-Malki et al. 2013; Aziza et al. 2019; Gong et al. 2021), SOD levels (SMD, 1.95; 95% CI, 1.3–2.6; *p* < 0.00001; heterogeneity: I^2^ = 77%) in 11 studies (Liu et al. 2006; Liu et al. 2009; Mansouri et al. 2011, 2015; Sayed 2012; Al-Malki et al. 2013; Bao et al. 2014; Salahuddin and Katary 2017; Gong et al. 2018; Ding et al. 2020; Gao et al. 2022), and CAT levels (SMD, 2.43; 95% CI, 1.4–3.45; *p* < 0.00001; heterogeneity: I^2^ = 80%) in 7 studies (Mansouri et al. 2011, 2015; Al-Malki et al. 2013; Bao et al. 2014; Salahuddin and Katary 2017; Gong et al. 2018; Ding et al. 2020) and reduced the MDA levels (SMD, −2.05; 95% CI, −2.67 to −1.44; *p* < 0.00001; heterogeneity: I^2^ = 73%) in 11 studies (Liu et al. 2006; Mansouri et al. 2011, 2015; Sayed 2012; Al-Malki et al. 2013; Bao et al. 2014; Salahuddin and Katary 2017; Gong et al. 2018, 2021; Ding et al. 2020; Gao et al. 2022).

#### Serum lipids: triglycerides, cholesterol, low-density lipoprotein (LDL), and high-density lipoprotein (HDL)

In DN animal models, the administration of PA was linked to improved serum dyslipidemia, including a reduction in triglycerides (SMD, −6.08; 95% CI, −8.58 to −3.59; *p* < 0.00001; heterogeneity: I^2^ = 83%) in 6 studies (Liu et al. 2009; Mansouri et al. 2015; Du et al. 2017; Wei et al. 2017; Gong et al. 2018, 2021), cholesterol (SMD, −3.49; 95% CI, −5.29 to −1.69; *p* < 0.00001; heterogeneity: I^2^ = 88%) in 6 studies (Liu et al. 2009; Mansouri et al. 2015; Du et al. 2017; Salahuddin and Katary 2017; Gong et al. 2018, 2021), and LDL (SMD, −4.92; 95% CI, −6.78 to −3.06; *p* < 0.00001; heterogeneity: I^2^ = 29%) in 2 studies. However, there was no effect on HDL.

#### Advanced glycation end products (AGE)

AGE analysis was included in 3 studies (Lee et al. 2007; Li et al. 2008; Li et al. 2009). The pooled results showed that PA significantly reduced AGE in DN animal models (SMD, −1.52; 95% CI, −2.14 to −0.90; *p* < 0.00001; heterogeneity: I^2^ = 0%).

#### Nuclear factor kappa B (NF-κB)

NF-κB analysis was included in 2 studies (Lee et al. 2007; Aziza et al. 2019). The pooled results revealed that PA significantly inhibited the activation of NF-κB in DN animal models (SMD, −3.53; 95% CI, −4.96 to −2.09; *p* < 0.00001; heterogeneity: I^2^ = 51%).

### Additional analysis and publication bias

Subgroup analysis based on animal species indicated that PA significantly reduced Scr in DN models of both rats (SMD, −2.25; 95% CI, −3.11 to −1.63; *p* < 0.00001; heterogeneity: *I*^2^ = 75%) and mice (SMD, −2.22; 95% CI, −4.10 to −0.35; *p* < 0.00001; heterogeneity: *I*^2^ = 83%). Based on the methods for establishing DN models, subgroup analysis showed that PA significantly reduced Scr in both STZ-induced (SMD, −2.38; 95% CI, −3.13 to −1.16; *p* < 0.00001; heterogeneity: *I*^2^ = 71%) and db/db (SMD, −2.22; 95% CI, −4.10 to −0.35; *p* < 0.00001; heterogeneity: I^2^ = 83%) DN models.

### Risk of bias

Overall, the detail of methodological quality was not mentioned in the included studies. Allocation concealment, random housing, blinding methods, and random outcome assessment were not stated in any of the included studies. All included studies were peer-reviewed publications and reported the basic characteristics of the animals. Sixteen studies reported the randomization of animals into the treatment or model group (Liu et al. 2006; Li et al. 2009; Mansouri et al. 2011, 2015; Sayed 2012; Bao et al. 2014; Du et al. 2017; Wei et al. 2017, 2018; Gao et al. 2018; Gong et al. 2018, 2021; Aziza et al. 2019; Ding et al. 2020; Lv et al. 2021; Gao et al. 2022). Incomplete outcome data and other biases were not detected in any of the studies. The study quality is summarized in Table 2.

**Table 2.** Summary risk of bias.

Study	A	B	C	D	E	F	G	H	I	J	Total
Al-Malki et al. (2013)	+	−	+	−	−	−	−	−	+	+	4
Aziza et al. (2019)	+	+	+	−	−	−	−	−	+	+	5
Bao et al. (2014)	+	+	+	−	−	−	−	−	+	+	5
Ding et al. (2020)	+	+	+	−	−	−	−	−	+	+	5
Du et al. (2017)	+	+	+	−	−	−	−	−	+	+	5
Gao et al. (2018)	+	+	+	−	−	−	−	−	+	+	5
Gao et al. (2022)	+	+	+	−	−	−	−	−	+	+	5
Gong et al. (2018)	+	+	+	−	−	−	−	−	+	+	5
Gong et al. (2021)	+	+	+	−	−	−	−	−	+	+	5
Lee et al. (2007)	+	−	+	−	−	−	−	−	+	+	4
Li et al. (2008)	+	−	+	−	−	−	−	−	+	+	4
Li et al. (2009)	+	+	+	−	−	−	−	−	+	+	5
Liu et al. (2006)	+	+	+	−	−	−	−	−	+	+	5
Liu et al. (2009)	+	–	+	−	−	−	−	−	+	+	4
Lv et al. (2021)	+	+	+	−	−	−	−	−	+	+	5
Mansouri et al. (2011)	+	+	+	−	−	−	−	−	+	+	5
Mansouri et al. (2015)	+	+	+	−	−	−	−	−	+	+	5
Salahuddin and Katary (2017)	+	−	+	−	−	−	−	−	+	+	4
Sayed (2012)	+	+	+	−	−	−	−	−	+	+	5
Wang (2016)	+	−	+	−	−	−	−	−	+	+	5
Wei et al. (2017)	+	+	+	−	−	−	−	−	+	+	5
Wei et al. (2018)	+	+	+	−	−	−	−	−	+	+	5

A: peer reviewed publication; B: presence of randomization of animals into treatment groups; C: basic characteristics; D: allocation concealment; E: random housing; F: blinding (caregivers and/or researchers); G: random outcome assessment; H: blinding (outcome assessor); I: incomplete outcome data; J: other sources of bias. ‘+’ indicates low risk of bias; ‘−’ indicates high risk of bias; and ‘?’ indicates an unclear risk of bias.

## Discussion

In this research, a systematic review and meta-analysis were performed to evaluate the efficacy and mechanisms of PA in DN animal models. A total of 22 studies involving 538 animals were included in this analysis. The pooled results asserted that treatment with PA significantly improved kidney function and proteinuria and reduced blood glucose levels. This review further elucidated that the protective effects of PA could be attributed to the anti-inflammatory, antioxidant, antifibrotic, and antiapoptotic effects; inhibition of endoplasmic reticulum (ER) stress; and alleviation of mitochondrial dysfunction and dyslipidemia.

**Figure 6.**
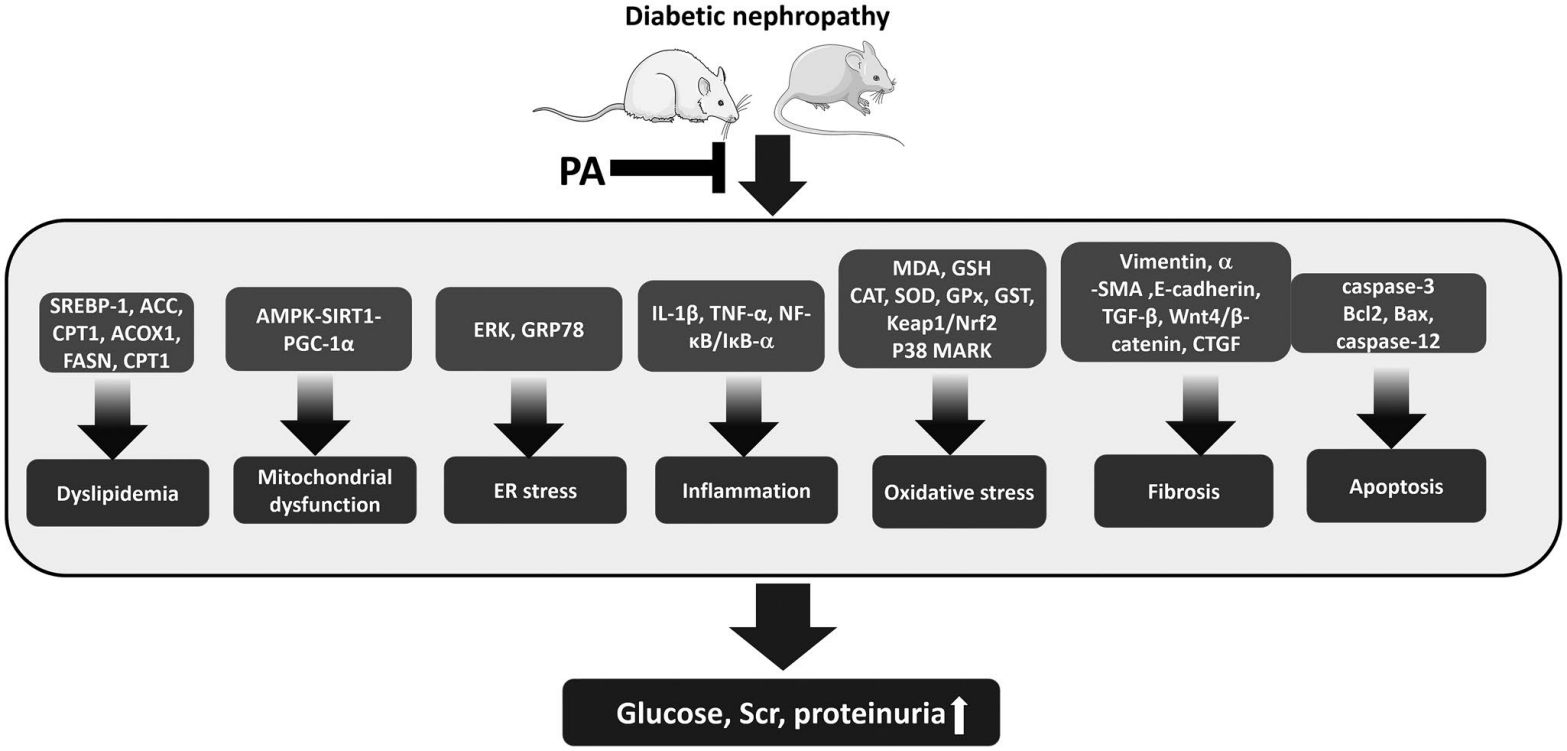
Protective mechanisms of PA on an animal model of diabetic nephropathy.

How PA protects against DN has not been completely elucidated. The mechanism of PA in DN in each study is illustrated in Table 3. The possible methods could be summarized as follows: (1) Anti-inflammatory effect: This review showed that PA exerted its anti-inflammatory effects by inhibiting the expression of interleukin-1β and tumor necrosis factor-α (TNF-α) and blocking the activation of NF-κB pathways. A study has reported that persistent inflammation of the renal tissue is involved in the development and progression of DN (Shang et al. 2019). Inhibition of TNF-α can decrease renal MDA levels and enhance renal SOD levels, thus improving renal mesangial expansion and tubular injury in DN rats (Cheng et al. 2021). Inflammatory cytokines can induce epithelial–mesenchymal transition and endothelial cell damage (Liu 2011; Platania et al. 2020), further resulting in ECM (Forbes et al. 2008) accumulation. In addition, inflammatory markers predict and precede the presence of proteinuria (Hofherr et al. 2022). NF-κB plays a key role in regulating inflammatory cytokines. Hyperglycemia can activate NF-κB and promote the transcription of chemokines and proinflammatory cytokines (Ahad et al. 2014). Inhibition of NF-κB improves kidney function by alleviating renal cortex inflammatory response and podocyte apoptosis in STZ-induced DN (Zhu et al. 2018). (2) Antioxidant effect: This review demonstrated that PA elevated SOD, GSH, CAT, glutathione S transferase, and glutathione peroxidase levels and decreased MDA levels in the kidney of DN animals. Furthermore, p38 MARK and Kelch-like ECH-associated protein 1 (Keap1)/nuclear factor erythroid 2-related factor 2 (Nrf2) pathways were activated. Nrf2 is one of the potent antioxidant molecules (Calabrese et al. 2010; Catino et al. 2015; La Rosa et al. 2020). The Nrf2 signaling network is implicated in neurological disorders as well as DN (La Rosa et al. 2020). The mechanisms of oxidative stress in DN are complex. Oxidative stress directly causes damage to endothelial cells, podocytes, and mesangial cells, which results in renal fibrosis and proteinuria in DN. Furthermore, oxidative stress indirectly causes kidney damage by activating pathogenic molecules, such as Ang-II and transforming growth factor-β1 (Forbes et al. 2008). Studies have reported that the inhibition of oxidative stress improves mitochondrial membrane integrity and attenuates mitochondrial damage and renal cell apoptosis in DN (Chen and Fang 2018). (3) Antifibrotic effect: PA exhibits an antifibrotic action on DN, as evidenced by inhibiting the expressions of α-SMA, vimentin, and twist-related protein 1 and promoting the expression of E-cadherin. Renal fibrosis is a common feature in the late stage of DN. The activation of TGF-β1, connective tissue growth factor, and Wnt4/β-catenin has been demonstrated to be involved in high glucose-induced renal fibrosis (Ren et al. 2019). (4) Antiapoptotic effect: In DN, hyperglycemia promotes and induces apoptosis in various types of kidney cells, such as podocytes and tubular epithelial cells, which results in the loss of kidney function (Adeghate 2004). Podocyte apoptosis plays a pertinent role in the development of proteinuria, whereas that of tubular epithelial cells leads to tubulointerstitial fibrosis. The inhibition of apoptosis exerts a significant protective effect in DN. Kidney cell apoptosis has been noted in the renal biopsies of patients with DN (Sifuentes-Franco et al. 2018). PA exerts its antiapoptotic effects in DN animals by downregulating B-cell leukemia/lymphoma 2 protein (Bcl-2)-associated protein x (Bax), caspase-3, and caspase-12 and upregulating Bcl-2 (Wei et al. 2018; Gao et al. 2022). (5) Inhibition of ER stress: High glucose levels can induce ER stress in podocytes and mesangial cells (Cao et al. 2014). ER stress can, in turn, lead to proteinuria by causing podocyte damage and ECM overproduction by inducing the proliferation of mesangial cells (Fan et al. 2017). An included study observed that PA can inhibit ER stress markers in DN animals (Gao et al. 2018). ER stress-induced renal cell apoptosis contributes to the development of kidney injury in STZ-induced DN (Liu et al. 2008). (6) Improvement of mitochondrial dysfunction and dyslipidemia: Mitochondrial dysfunction occurs in the early stage of DN and impairs respiratory chain function. Subsequently, it disrupts the balance in the production and utilization of ATP in kidney cells, which results in increased oxygen consumption and renal hypoxia (Wei and Szeto 2019). Dysfunctional mitochondria also disrupt cellular signaling and aggravate reactive oxygen species generation, which accelerates the progression of DN (Forbes and Thorburn 2018). Dyslipidemia has been demonstrated to accelerate renal damage in patients with diabetes (Palazhy and Viswanathan 2017). Moreover, dyslipidemia is associated with increased cardiovascular events in such patients (Chen and Tseng 2013). This review proved that PA could improve mitochondrial dysfunction and dyslipidemia in DN animals. Furthermore, it sheds light on the complex mechanisms by which PA offers protection against DN. However, which mechanism is more important should be studied further. The protective mechanism of PA in DN is summarized in Figure 6.

**Table 3.** Summary of mechanism of PA on protecting diabetic nephropathy mice.

Study	Model		Effect	Upregulation	Downregulation
Al-Malki et al. (2013)	STZ and high-fat diet induced diabetic nephropathy		Reduce glucose; anti-oxidative stress; anti-inflammation; inhibition kidney mesangial expansion; anti-fibrosis	GSH; CAT; Glutathione reductase; GPx;	NF-κB; IL-6; TGF-β1; RAGE
Aziza et al. (2019)	STZ induced diabetic nephropathy		Reduce glucose; improve kidney function; anti-oxidative stress	SOD; GSH	NF-κB; MDA
Bao et al. (2014)	STZ and high-carbohydrate/high-fat diet induced diabetic nephropathy	Low dose	–	–	–
		Medium dose	Improve β-cell function; improve mitochondrial dysfunction	Nephrin; podocalyxin; PARP; RAGE; TFAM; NRF1; AMPK/SIRT1/PGC-1α	–
		High dose	Reduce glucose; reduce proteinuria; anti-oxidative stress; improve β-cell function; improve mitochondrial dysfunction	Nephrin; podocalyxin; SOD; CAT; RAGE; PARA; TFAM; NRF1; AMPK/SIRT1/PGC-1α	MDA
Ding et al. (2020)	STZ, high-sugar and high-lipid fodder induced diabetic nephropathy		Insulin resistance; reduce glucose; improve kidney function; reduce proteinuria; anti-oxidative stress	SOD; T-AOC; GSH, Nrf2; OH-1; GST; NQO1	MDA
Du et al. (2017)	db/db diabetic nephropathy		Reduce glucose; improve kidney function; reduce proteinuria; improve dyslipidemia	PPARα; ACOX1; CPT1	SREBP-1; ACC; FASN;
Gao et al. (2018)	STZ and high-fat diet induced diabetic nephropathy		Reduce glucose; improve kidney function; inhibition kidney mesangial expansion; anti-apoptosis; inhibition endoplasmic reticulum stress		ERK; caspase-12; GRP78
Gao et al. (2022)	STZ induced diabetic nephropathy		Reduce glucose; improve kidney function; improve kidney tubular damage; anti-oxidative stress	SOD; Wnt4; β-catenin	MDA
Gong et al. (2018)	STZ induced diabetic nephropathy		Reduce glucose; improve dyslipidemia; improve kidney morphological damage	CAT;	MDA; PCO
Gong et al. (2021)	Cdcl_2_, high-fat and high-sugar diet induced diabetic nephropathy		Reduce glucose; improve kidney function; improve dyslipidemia; improve tubulointerstitial fibrosis and glomerular damage	Nrf2; SOD; GSH; GST	MARK; p38; Keap1; NO; MDA; PCO;
Lee et al. (2007)	STZ induced diabetic nephropathy	Polymeric PA	–	–	–
		Oligomeric PA	Reduce glucose; improve kidney function; reduce proteinuria; anti-oxidative stress	GSH; IκB-α	COX-2; iNOS; ROS; TBARS; NF-κB
Li et al. (2008)	STZ induced diabetic nephropathy		Reduce glucose; improve kidney function; improve kidney morphological damage; anti-oxidative stress	GST; NADH-ubiquinone oxidoreductase; SBP2; GCP; F1-ATPase beta subunit; LOC500183 protein	AGEs; GSTM; AFAR; Resiniferatoxin-binding, phosphotriesterase-related protein
Li et al. (2009)	STZ induced diabetic nephropathy		Reduce glucose; improve kidney function; reduce proteinuria; improve kidney morphological damage	–	AGEs; CTGF
Liu et al. (2006)	Alloxan induced diabetic nephropathy		Improve kidney function; reduce proteinuria; anti-oxidative stress	SOD; T-AOC; NO; NOS	MDA
Liu et al. (2009)	STZ and high-fat diet induced diabetic nephropathy		Reduce glucose; improve dyslipidemia; anti-oxidative stress	SOD;	NO; NOS
Lv et al. (2021)	STZ and high-fat diet induced diabetic nephropathy		Reduce glucose; improve kidney function; reduce proteinuria; anti-fibrosis	E-cadherin	Vimentin; TWIST1
Mansouri et al. (2011)	STZ induced diabetic nephropathy		Reduce proteinuria; anti-oxidative stress	SOD; GPx; CAT	MDA
Mansouri et al. (2015)	STZ induced diabetic nephropathy		Reduce glucose; improve kidney function; reduce proteinuria; improve dyslipidemia; anti-oxidative stress	SOD; GPx; CAT	MDA
Salahuddin and Katary (2017)	STZ induced diabetic nephropathy		Reduce glucose; improve kidney function; reduce proteinuria; improve dyslipidemia; anti-oxidative stress; anti-inflammation	GPx; SOD; CAT	IL-6; TNF-α; CRP; MDA
Sayed (2012)	STZ induced diabetic nephropathy		Reduce glucose; improve kidney function; anti-oxidative stress; anti-inflammation	GSH; SOD	NO; IL-6; MDA
Wang 2016	STZ induced diabetic nephropathy		Reduce glucose; improve kidney function; reduce proteinuria		NO; GLUT2
Wei et al. (2017)	db/db diabetic nephropathy		Reduce glucose; improve kidney function; reduce proteinuria; anti-fibrosis	E-cadherin	α-SMA; ERK; MARK; p38
Wei et al. (2018)	db/db diabetic nephropathy		Reduce glucose; improve kidney function; reduce proteinuria; anti-apoptosis	Bcl-2	Bax; caspase-3; ERK; MARK; p38; cytochrome c; TXNIP

PA has been successfully translated from preclinical studies to clinical trials in certain diseases and has shown therapeutic effects. Treatment with PA for 1 year can significantly alleviate retinal thickening with hard exudates in patients with diabetic retinopathy (Moon et al. 2019). Another clinical study noted that PA might prevent the recurrence of symptomatic urinary tract infections in women (Babar et al. 2021). The study showed that PA is effective in assuaging menopausal symptoms and reducing blood pressure in middle-aged women (Terauchi et al. 2014). Regarding the safety of PA in humans, a clinical study to determine its safety and tolerability in healthy Japanese individuals, and the results suggested that the oral intake of up to 2,500 mg of PA for 4 weeks was safe and well tolerated by the participants (Sano 2017). However, the effect of PA in patients with DN has not been assessed in clinical studies. This review provides preclinical evidence that PA is a promising drug for treating DN. Nonetheless, challenges exist in its clinical translation for the treatment of DN. First, suitable doses of PA and the treatment duration for patients with DN are yet to be determined. Second, its side effects in humans have not been adequately studied. To ensure the quality of clinical trials, we recommend that a randomized placebo-controlled trial of PA on patients with DN be conducted in the future.

There are certain limitations in this study. First, significant heterogeneity was observed in the primary outcomes and certain secondary outcomes, which indicates the instability of the pooled results. This high heterogeneity could be attributed to variations in the animal species, PA doses, and the methods to establish DN animal models. To address this heterogeneity, subgroup analysis was performed based on animal species and the methods to create the DN models, which reduced the heterogeneity only slightly. Therefore, heterogeneity might have resulted from other sources. More subgroup analyses must be performed to address this limitation when additional studies are conducted in the future. Second, the quality of the included studies was relatively low. To address this issue, we suggest that an animal study registry be used to augment the study quality. A scientific and rigorous research protocol must be established before commencing the experiment, and experimental information, such as allocation concealment, random housing, blinding methods, and random outcome assessment, must be recorded in it.

## Conclusions

This study established that PA can improve kidney function in DN animals. The protective effect of PA in DN are associated with its anti-inflammatory, antioxidant, antifibrotic, and antiapoptotic effects; inhibition of ER stress; and alleviation of mitochondrial dysfunction and dyslipidemia. This preclinical systematic review showed that PA might be a promising drug for the treatment of patients with DN.

## Data Availability

Data will be made available on request.
